# Higher cost, comparable outcomes: A health economic evaluation of patient‐specific instrumentation vs. conventional instrumentation for total knee arthroplasty in a Dutch aging population

**DOI:** 10.1002/jeo2.70447

**Published:** 2025-10-27

**Authors:** Isobel M. Dorling, Dieuwertje M. J. Theeuwen, Ghislaine A. P. G. van Mastrigt, Tim J. M. Welting, Martijn G. M. Schotanus, Bert Boonen

**Affiliations:** ^1^ Department of Orthopaedic Surgery Zuyderland Medical Center Geleen Netherlands; ^2^ CAPHRI, Care and Public Health Research Institute Maastricht University Maastricht Netherlands; ^3^ Department of Orthopaedic Surgery and Research Maastricht University Medical Center Maastricht Netherlands

**Keywords:** cost‐effectiveness, Dutch aging population, health economics evaluation, patient‐specific instrumentation, total knee arthroplasty

## Abstract

**Purpose:**

The demand for treatment of knee osteoarthritis is rising as the population ages. To optimise outcomes of total knee arthroplasty (TKA), several innovative techniques have been introduced, including patient‐specific instrumentation (PSI). While these developments may improve clinical outcomes, they are often associated with higher healthcare costs. The present study aimed to evaluate the long‐term cost‐utility of PSI compared to conventional instrumentation (CI) for TKA, from a hospital perspective.

**Methods:**

A multicenter randomised controlled trial including 180 patients was conducted in 2010, with 45 patients in each treatment arm per center. For the current economic evaluation, data from one participating center (*n* = 90) were analysed. Patients were assigned to receive either PSI or CI TKA and completed EQ‐5D‐5L questionnaires preoperatively and at 5‐ and 10‐year follow‐up. Utility scores and Quality‐Adjusted Life Years (QALYs) were derived from these questionnaires. Hospital records provided cost data, which were indexed to 2023 values. Incremental cost‐effectiveness ratios (ICERs) were calculated as cost per QALY gained.

**Results:**

At final follow‐up, 23 patients (26%) were lost due to death (*n* = 15, 17%) or withdrawal (*n* = 8, 9%). The cohort included 58% females with a mean age of 67 years. QALY gains did not differ significantly between groups at either follow‐up point. Mean total costs were €10,722.58 per patient for PSI and €10,063.89 for CI, with PSI exceeding CI by €658.69. This translated into an ICER of €14,856.07/QALY after 5 years and €12,836.66/QALY after 10 years for PSI, compared to €13,932.24/QALY and €12,550.40/QALY, respectively, for CI.

**Conclusion:**

Both PSI and CI TKA improved quality of life at 5 and 10 years postoperatively. However, PSI incurred higher costs without demonstrating additional clinical benefit. From a hospital perspective, CI remains the more cost‐effective approach.

**Level of Evidence:**

Level II, retrospective study of an RCT.

**Abbreviations**
3D: three‐dimensional
BMI: body mass index
Ci: confidence interval
Ci: confidence interval
ICER: incremental cost‐effectiveness ratio
ICUR: incremental cost‐utility ratio
MCAR: missing completely at random
MRI: magnetic resonance imaging
NE: north east
NW: north west
OR: operating room
PSI: patient‐specific instrumentation
SD: standard deviation
SE: sout east
SW: south west
TKA: total knee arthroplasty

## INTRODUCTION

Total knee arthroplasty (TKA) is one of the most commonplace surgeries performed worldwide, with over 1 million cases performed in 2021 [10]. The number of cases per year was predicted to increase and had increased over the past decade [27]. In the Netherlands, the burden of hip and knee osteoarthritis (2015–2017) was estimated to be € 26.9 million based specifically on sick leave within the workforce [1, 13]. The direct medical costs for osteoarthritis in the Netherlands was estimated at € 1.1 billion in 2019 [19]. These numbers have led to preventative measures being undertaken, such as education on lifestyle related factors of osteoarthritis from a government level. However, the increase of surgical intervention has also been observed with 42.000 hospitalisations for osteoarthritis in 2020 [18]. Not only in the Netherlands but also in the United States a massive increase of TKA has been noted. Epidemiologic analyses of the United States showed that the increase for TKA is expected to be 139% by 2040 and 469% by 2060 [27]. The demand for TKA thus remains on the rise.

Historically, dissatisfaction rates up to 20% have been reported for TKA [8]. Recent evidence, however, suggests that this rate is closer to 10% [8]. These rates have led orthopaedic surgeons and researchers to strive for optimal and accurate placement of TKA, to improve clinical outcome [25]. To optimise prosthetic alignment, patient‐specific instrumentation (PSI) has been introduced for TKA [25]. The theory behind PSI was that a 3‐dimensional (3D) reconstruction was made of the knee and subsequently a 3D‐printed jig was produced, to use intraoperatively as an alignment tool. This tool would ensure more accurate alignment and therefore improved clinical results. However, this advancement has demonstrated no significant difference in clinical outcomes when compared to conventional instrumentation (CI) for TKA [23, 31]. Most new technological developments for TKA add additional costs to the procedure, due to the additional required equipment. Nevertheless, if these developments ensure better clinical outcomes, despite their higher costs, they could be cost‐effective regardless. As these new technological improvements have emerged over the past decades, an increased interest in costs, cost‐effectiveness and cost‐utility has been noted. Many studies have investigated differences in monetary costs of PSI and CI, with differing results [9]. Most studies conclude that PSI is more costly compared to CI, mainly due to the manufacturing of the 3D‐printed jigs used intraoperatively, and the magnetic resonance imaging (MRI) necessary for jig production. Notwithstanding, PSI has proven to be more efficient with decreased operating room (OR) time [4] and decreased operating tray usage [2]. These differences influence the procedure costs. Currently, no long‐term studies directly evaluate the cost‐effectiveness of PSI with CI TKA.

The hypothesis of this study was that PSI for TKA is more costly than CI for TKA due to additional manufacturing and imaging costs, despite less OR time and tray usage. The expected effect of the interventions was hypothesised to be similar, as clinical studies have shown no clinical significant difference in patient reported outcome measures for both techniques [5, 26]. We therefore investigated the difference in cost‐effectiveness of PSI TKA versus CI TKA 5‐ and 10‐years postoperatively, from a hospital perspective.

## MATERIALS AND METHODS

### Study design and participants

In 2010, a randomised controlled trial (RCT) recruited a total of 180 patients, of which 90 at the Zuyderland Medical Centre, the Netherlands [5, 26]. This single center trial was registered under the ABR registration number: NL31469.096.10. The study was performed in accordance with the declaration of Helsinki, version 2008 [29]. In this study patients from our center were analysed: 45 patients received TKA using CI and 45 patients received TKA using PSI. The other center was excluded due to inaccessibility to their costing data.  The patients were followed up at the 1‐, 2‐, 5‐ and 10‐year mark.  The inclusion criteria of the primary study were as follows: patients with disabling osteoarthritis, that were candidates for unilateral TKA [5]. The exclusion criteria included: patients with metal near the knee, ankle or hip joint; patients unable to undergo MRI‐imaging, and patients who had previously undergone knee surgery (excluding arthroscopic meniscectomy) [5]. The baseline characteristics of the original study included: male/female gender (40/50, *p*‐value n.s.), mean age (65 years, standard deviation [SD] 8.8, *p*‐value 0.001), and mean body mass index (29.5, *p*‐value n.s.) [5].   At the time of study conduction, no in‐trial economic analysis was performed. Thus, the current study was performed retrospectively from a hospital perspective. Earlier follow‐up dates were not included in the study, as the quality‐of‐life results could have been affected by ongoing recovery and difference in effect were not expected based on the study results. For this current study, the CHEERS 2022 reporting standard and guidance for health economic evaluations was used [6].

### Randomisation, blinding and data collection

Randomisation was computer generated. More detailed information on the randomisation process can be found in the original article [5]. The patients as well as the researchers were blinded to the type of intervention; at the 2‐year follow‐up patients were unblinded. Preoperatively and 1‐, 2‐, 5‐, and 10‐years postoperatively the patients received EQ‐5D‐5L (Dutch tariff) questionnaires to assess quality of life after TKA [12]. These were filled in at the outpatient clinic or sent by post. The forms were entered into the electronic data capture system.

### Outcomes

For the cost‐effectiveness analysis, the EQ‐5D‐5L was used as the primary outcome. This is a standardised instrument which assesses five different aspects of health [12]. These aspects include mobility, self‐care, usual activities, pain/discomfort, and anxiety/depression. These five aspects can be scored as no‐, slight‐, moderate‐, severe‐, and extreme‐ problems/unable to do. Additionally, the patients had to score their overall health from 0 to 100, with 0 meaning the worst imaginable health and 100 meaning the best imaginable health. The estimated benefit from the EQ‐5D‐5L was transformed into the number of gained or lost QALYs by calculating the index values [18]. These values ranged from 0 to 1.000, with 0 being death and 1.000 being perfect quality of life. Patients lost to follow‐up due to death thus received the utility 0. The index values can be calculated using an online tool [14]. These index values were used to calculate the delta quality‐adjusted life year (Δ QALY) during the follow‐up years. This was calculated by subtracting the utility measured preoperatively with the utility measured during the different follow‐up years, and dividing this value by the number of years it is counted over. By doing this, the mean gain or loss in utility was determined over the course of 1 year. It was assumed that the changes over this time period were linear.

### Costs

The analysis was performed from a hospital perspective. All costs were presented in euros (€). 2023 was used as the reference year.

The calculation of costs related to the surgery were split into three separate categories: preoperatively, intraoperatively and postoperatively.

Preoperative costs included costs of initial presentation at the outpatient clinic, standard X‐ray imaging and/or MRI (for the PSI group), production of the PSI mold, preoperative health assessment, preoperative lab work, and preparation of the surgical trays.

For the intraoperative category, all relevant data correlating to the surgery was calculated. This included the knee prosthetic itself, OR usage costs and time. The OR time cost was not available in our center. The pricing for OR time was therefore based on the Dutch reference pricing [16].

The postoperative costs were attributed to standard postoperative care (a single X‐ray of the knee the first day after surgery, and a personal session with a physiotherapist), length of hospital stay costs and revision surgery during the follow‐up. The Δ costs were calculated for each separate category. The Δ total cost were calculated for all abovementioned cost categories together. To preserve the costing integrity of our center, an indication of costs based on the reference prices of healthcare costs in the Netherlands were presented in Table 1. These were available from the Dutch manual for costs for economic evaluation in the healthcare sector: methodology and references prices, version 2024 [16].

**Table 1:** An overview of costs retrieved from the publicly available online manual for reference pricing in the Netherlands in euros [16].

Cost category	Reference price in the Netherlands in euros (€)
Intake at outpatient clinic	€ 120
MRI of the knee	€ 264
Standard X‐ray of the knee	€ 82.17
Costs of OR time, defined per minute	€ 11.09
Costs of admittance in the hospital, defined per day	€ 644

*Note*: Not all of the costs as described in the materials and methods are given in this table. Only available costs from the manual. The actual costs of our center were not given to ensure integrity. The costs given in this table are indicative of costs in our center.

Abbreviations: MRI, magnetic resonance imaging; OR, operating room.

Specific, detailed data were not available on the costs of healthcare staff, such as cooking‐ and cleaning staff, residents, specialists and nurses. These were not taken into account when performing the current analysis.

### Analysis of data

ICER was calculated by subtracting the mean total costs of the PSI group from the CI group, divided by the mean QALY of the PSI group subtracted from the CI group (ΔTotal Cost/Δ QALY) [18].

For the cost‐effectiveness analysis, discount rates of 3% for costs and 1.5% for effects were applied in accordance with Dutch guidelines for economic evaluations [22]. The ICER was calculated for base case (with and without discounting), the upper and lower bounds of the confidence intervals and worst‐ and best‐case scenarios for both follow‐up periods. To emulate a best‐ and worst‐case analysis, either +10% or −10% cost and either +10% or −10% QALY gain was applied for PSI and CI.

ICER is interpreted with the cost‐effectiveness plane [3]. This plane is divided into four quadrants: northwest (NW), northeast (NE), southwest (SW), southeast (SE). These quadrants represent: increased cost with less effect (NW), increased cost with more effect (NE), decreased cost and less effect (SW), decreased cost and more effect (SE). The National Institute for Health in the Netherlands (ZorgInstituut Nederland) advises a cost‐effectiveness threshold of up to €50,000 per QALY gained for health interventions related to osteoarthritis based on a proportional shortfall of 0.41 [32]. Therefore, any ICER that falls under the threshold is regarded as cost‐effective within the cost effectiveness plane. In addition, a sensitivity analysis was performed by calculating the ICER when comparing PSI TKA to CI TKA. No subgroup analysis was performed.

### Statistical analysis

All data was analysed using IBM SPSS version 29. To analyse the effect of missing data on QALY and cost/QALY analysis, the ‘missing complete at random' (MCAR) test was used [13]. If data was MCAR, mean imputation was used due to normally distributed data. A significance level of *p* ≤ 0.05 (95% confidence interval (Ci)) was used to determine significance. As quality of life was not normally distributed, a non‐parametric bootstrap sampling method with 1000 replications was used to assess statistical significance of the differences between the two treatment groups. Bootstrapping was not used for the cost outcomes as the hospital perspective of this analysis ensured fixed costs which are linear during the follow‐up period.

## RESULTS

### Baselines

**Figure 1:**
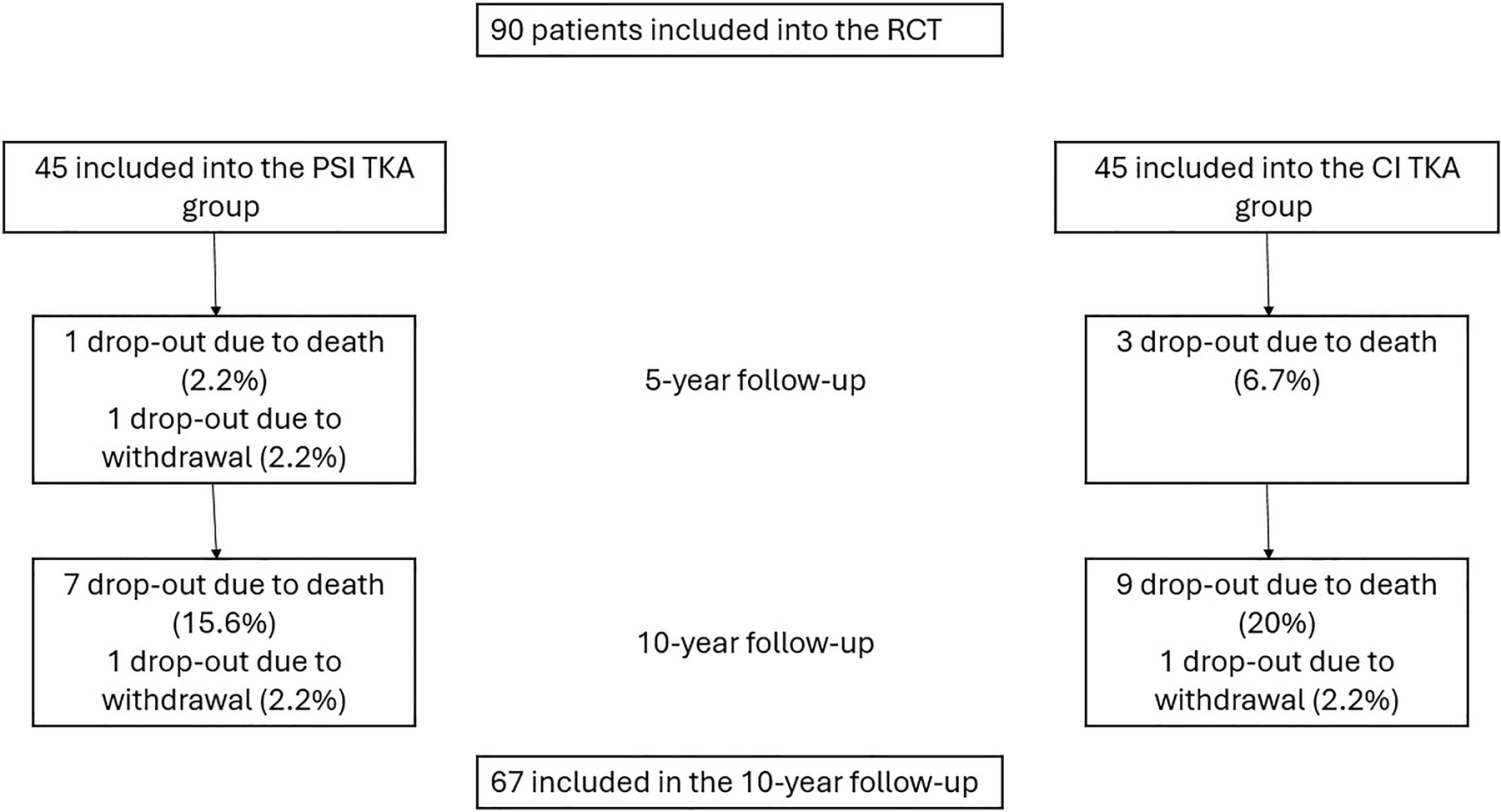
Patient drop‐out rates during the 10‐year follow‐up period.  All reported deaths were not related to the study intervention. CI, conventional instrumentation; PSI, patient‐specific Instrumentation; TKA, total knee arthroplasty.

At baseline, among the 90 included in the study, 58% patients were female. During the follow‐up, 23 patients (26%) were lost to follow‐up due to death (*n* = 15, 17%) or withdrawal (*n* = 8, 9%). The mean age was 69.2 years for the PSI group and 65.4 years for the CI group. Figure 1 displays a flowchart of the follow‐up of patients during the past 10 years. Table 2 displays the baseline characteristics at time of inclusion.

**Table 2:** Baseline characteristics patient population at time of inclusion.

	PSI (*n* = 45)	CI (*n* = 45)	Significance between groups (*p*‐value)
Age (years)	69.2 (SD ± 8.53, 95% Ci 66.6–71.7)	65.4 (SD ± 9.6, 95% Ci 62.5–68.3)	0.512
Height (m)	1.67 (SD ± 0.08, 95% Ci 1.65–1.70)	1.70 (SD ± 0.09, 95% Ci 1.67–1.73)	0.072
Weight (kg)	83.3 (SD ± 11.9, 95% Ci 79.7–86.9)	81.9 (SD ± 14.5, 95% Ci 77.5–86.3)	0.663
BMI (kg/m^2^)	29.4 (SD ± 4.0, 95% Ci 28.3–30.7)	28.2 (SD ± 3.7, 95% Ci 27.1–29.3)	0.062

*Note*: The means of following outcomes were calculated for the cohort, at time of inclusion: age in years, height in metres, weight in kg and BMI in kg/m^2^. The results are presented as means with standard deviations (±) and 95% confidence intervals (Ci). Statistical significance was determined at a *p*‐value of *p* ≤ 0.05. No statistically significant differences were found at baseline, as demonstrated in this table.

Abbreviations: BMI, body mass index; Ci, confidence interval; CI, conventional instrumentation; PSI, patient‐specific Instrumentation.

### Missing data

Little's MCAR test showed a significance of *p* = 0.096 for missing data of the EuroQol‐5D for the entire follow‐up period. The missing data was random.

### Quality of life

A complete overview of the mean QALY gain for the total population, PSI group, and CI group after 5‐ and 10‐years can be found in Table 3. The table includes SDs and 95% Ci. All mean utilities and significance thereof are reported in the original studies and corresponding follow‐up studies [4, 5, 26, 28].

**Table 3:** Overview of QALY gain after PSI and CI TKA.

	Total QALY gain (PSI and CI)		QALY gain PSI		QALY gain CI		*p* value
5‐Years postoperative	0.72 (±0.48, 95% Ci 0.62–0.82)	*n* = 80	0.72 (±0.53, 95% Ci 0.56–0.88)	*n* = 37	0.72 (±0.44, 95% Ci 0.59–0.85)	*n* = 43	0.308
10‐Years postoperative	0.82 (±1.19, 95% Ci 0.56–1.06)	*n* = 67	0.83 (±0.86, 95% Ci 0.57–1.09)	*n* = 32	0.80 (±1.46, 95% Ci 0.36–1.24)	*n* = 35	0.166

*Note*: The QALY gain for the total group, for the PSI group and the CI group is given 5‐ and 10‐years postoperatively. The results are presented as means with standard deviations (±) and 95% confidence intervals (Ci). Additionally, the number of patients (*n*) is given per group 5‐ and 10‐years postoperatively, as well as the *p*‐value.

Abbreviations: CI, conventional instrumentation; Ci, confidence interval; PSI, patient‐specific instrumentation.

No statistically significant differences were found between PSI and CI TKA for the amount of gained QALYs/year, 5‐ and 10‐years postoperatively (*p* ≥ 0.05). The data is additionally presented in Table 3 with SDs and Cis.

### Total costs

For PSI TKA, the total costs were €10,722.58 per patient. For CI TKA, the total costs were €10,063.89 per patient. In total, the usage of PSI TKA costs €658.69 more than CI TKA per patient. Total costs are the standard costs for the entire pathway of care; these do not differ at the 5‐ and 10‐year follow‐up period.

### Preoperative costs

These costs included the costs of standard pre‐operative workup, imaging, mold production.  All preoperative costs combined amounted to €971.66 less for CI TKA.

### Intraoperative costs

The total OR cost and cost of the prosthetic itself is equal for PSI and CI TKA. This amounts to €0 difference between the two techniques. The main difference between the two techniques intraoperatively is the OR time and tray use. The number of surgical trays used for PSI TKA were five large trays and four small trays. For CI TKA, seven large trays and three small trays were used. The difference in cost for the sterilisation of these trays is €132.93 more for CI TKA. From the cohort of patients, the reduced OR time for PSI TKA was a mean of 11 min per case. OR costs were defined as €11.09 per minute [4]. This amounted to a difference of €254.92 less for PSI TKA for intraoperative costs.

### Postoperative costs

These costs included the costs of the hospital stay and standard postoperative care. The mean length of hospital stay for PSI TKA was 4.3 days. The mean for CI TKA was 4.4 days. This amounted to a total postoperative cost of €58.05 less for PSI TKA.

### Revision rates

No difference was found in the number of revisions performed between the two groups. For the CI group one revision was reported due to lateral knee pain. For the PSI group one revision was performed due to aseptic tibial plateau loosening. Both patients received revision in the first year after surgery. Revisions were accounted for in the sensitivity analysis by adding their costs to the initial costs of care.

### Cost‐utility

With discounting, the cost‐utility measure for PSI TKA was €13,839.20/QALY gained after 5‐years and €11,156.02/QALY gained after 10‐years. With discounting the cost‐utility measure for CI TKA was €12,989.06/QALY gained after 5‐years and €10,863.36/QALY gained after 10‐years.

### ICER and sensitivity analysis

**Figure 2:**
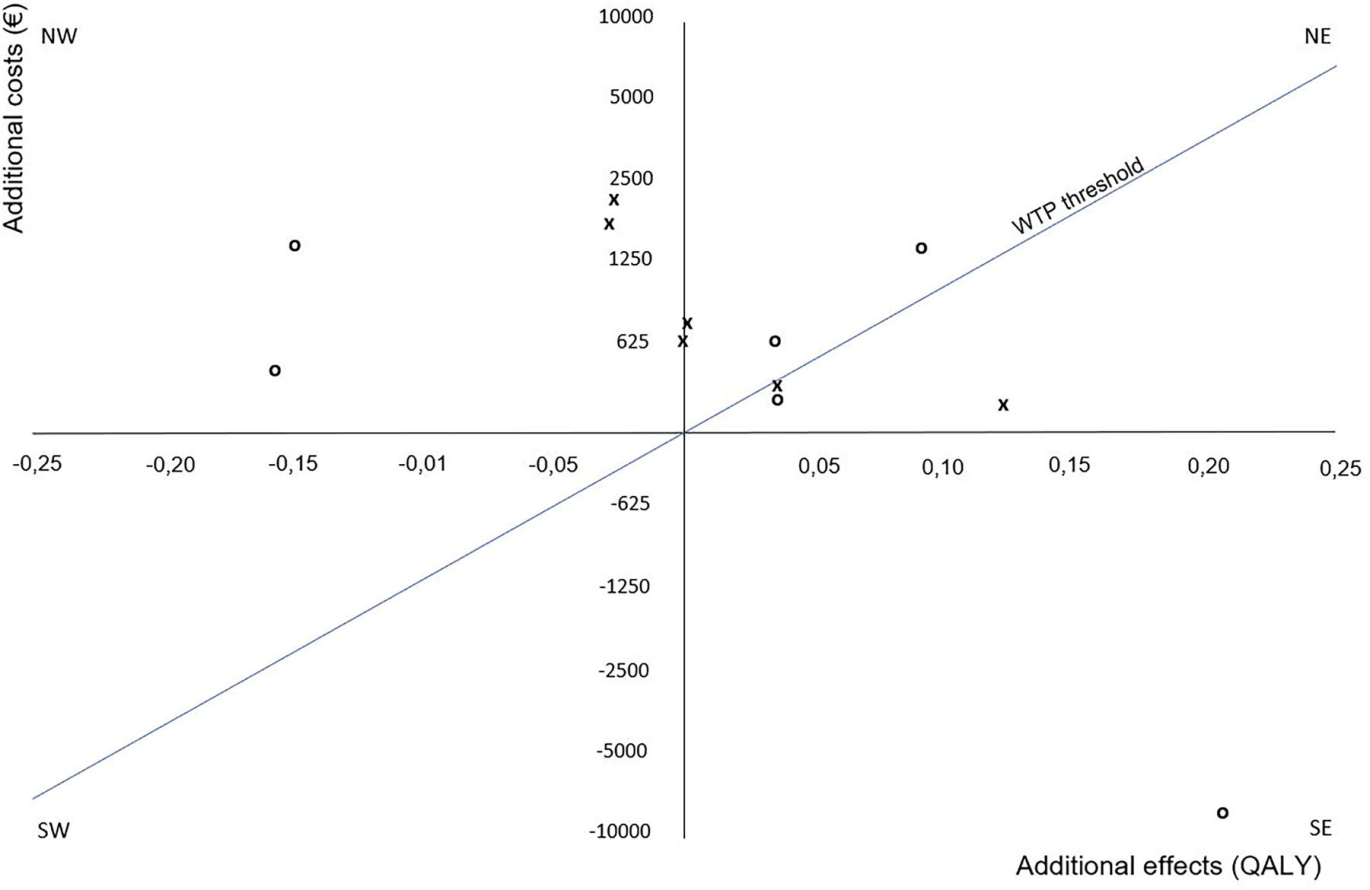
Cost‐effectiveness plane comparing PSI TKA to CI TKA in different scenarios.  This figure shows a scatter plot of the sensitivity analysis including discounting scenarios, upper‐ and lower bounds and the best‐ and worst‐case scenarios when comparing PSI to CI TKA in a timeframe of 5‐ and 10‐years postoperatively. The plot compares additional costs in different scenarios to different effects (QALY). The plot shows multiple scenarios under the WTP threshold (€50.000).   All scenarios (base case as well as best‐ and worst‐ case) in the 5‐year follow‐up are indicated by ‘x’. All scenarios in the 10‐year follow‐up are indicated by ‘o’. CI, conventional instrumentation; NE, northeast; NW, northwest; PSI, patient‐specific instrumentation; QALY, quality adjusted life year; SE, southeast; SW, southwest; TKA, total knee arthroplasty; WTP, willingness to pay.

A sensitivity analysis was performed by calculating the ICER when comparing PSI TKA to CI TKA. As utilities were not significantly different in all scenarios, ICUR was not calculated in the current study. The analysis, as illustrated in Figure 2, delineates the positioning of the calculations across the various quadrants of the cost‐effectiveness plane.

Base case analysis showed that PSI is more expensive and more effective after 5‐ and 10‐years, situating in the NW of the cost‐effectiveness plane in Figure 2. This indicates a higher cost with a higher QALY gain. The sensitivity analysis showed that the cost‐effectiveness of PSI improves if QALY gains are higher but worsens if clinical outcomes worsen. The cost‐effectiveness of CI remains favourable; however, it contains considerable variability in the sensitivity analysis, attributable to fluctuations in the estimated effect bounds. In other words, if CI performs better or worse clinically, it has more effect on the cost‐effectiveness than that it would for PSI. This indicates that the cost‐effectiveness of CI is highly sensitive to postoperative quality‐of‐life outcomes obtained after 5‐ and 10‐years.

This analysis showed that in the best‐case both PSI and CI showed improved cost‐effectiveness, so both situating in the NE quadrant of Figure 2. CI showed an increased cost‐effectiveness than PSI in the best case. In the worst case both interventions present a decrease in cost‐effectiveness, situated in the NW quadrant, but more for PSI when compared to CI. True cost‐effectiveness is situated in the SE quadrant. If CI performs at its lowest bounds, PSI could hypothetically be situated here after 10‐years.

## DISCUSSION

The aim of the present study was to compare cost‐effectiveness of PSI and CI TKA. The main finding was that PSI TKA costs €658.69 more per procedure than CI TKA if all applicable costs, from a hospital perspective, are considered. PSI TKA was less cost‐effective after 5‐ and 10‐years.

Comparable literature is scarce, but the current presented results are reflective of other results published in Europe. Räsänen et al. presented a cost per QALY of €13,995, 1 year after primary CI TKA [21]. Furthermore, Schilling et al. presented their estimate of 0.77 of QALY gain 7‐years after CI TKA in 2016 [21, 24]. This data on cost per QALY and QALY gain 5‐ to 10‐years after primary TKA corresponds to the presented data which was derived from our center.

Cost‐effectiveness also depends on the willingness to pay threshold. In the Netherlands the threshold is up to €50,000 per QALY gained for health interventions related to osteoarthritis [32]. In the current study the cost per QALY for both PSI and CI TKA are within this range and could therefore both be accepted as cost‐effective interventions. Additionally, the ICER 10‐years after PSI falls within this range, and therefore PSI could be considered cost‐effective after 10‐years. Reports have shown that PSI TKA can be a useful tool in anatomically challenging cases. Mainly, in cases where intramedullary alignment tool use is not possible or when there are posttraumatic anatomical changes [17]. The PSI method offers surgeons an alternative guidance option. The increased cost per patient case could therefore have its value in specific situations.

This study has a few limitations. First, the cost‐effectiveness analysis was performed from a single hospital perspective. Unfortunately, calculations performed from this perspective do not offer insight into the effects of TKA after hospitalisation. The possible cost‐benefit of return to work, care burden and overall health‐benefits with improved mobility have not been taken into account in our analysis. Due to this, the current analysis did not give a full overview of the societal impact of the intervention. TKA has been shown to improve quality of life, with increased mobility and decreased pain being the main attributing factors [7, 20]. This in turn ensures increased return to work and sports of up to 90% one year postoperatively. This could have major cost‐effectiveness implications for all types of TKA, which should be the focus of future research. Another limitation is that the RCT on which this study was based, was performed in 2010. Although the clinical outcomes are expected to be similar now, this has an implication on the costs. For instance, the length of hospital stay in 2010 was 4.2 days, while it is currently 2 days due to pathway optimisation [11]. Additionally, change in the current pathways has impact on staff costs, which were not available for the current analysis as mentioned in the materials and methods. This impacts total costs and thus cost‐effectiveness. So, while costs were converted and discounted from 2010 to 2023, the current analysis might give a slight skewed view on the total cost outcome.

Furthermore, the hospital costs were derived from a single center which makes this analysis site‐specific. Secondly, as patients age the EQ‐5D utility scores are expected to worsen linearly [30]. As a result, the present analysis could include outliers in utility scores which are not attributed to the TKA, but to overall decline in perceived health [15].

The strength of this analysis was that it presents data which was collected over the course of 10‐years. As of now, no cost‐utility analysis over this period of time is available for PSI and/or CI TKA. Additionally, the data was missing completely at random, which indicates that there is little bias within calculated QALYs. Lastly, the analysis aimed to look at many different variables that influence costs within the hospital, to create valid evidence based on a single hospital setting.

## CONCLUSION

PSI and CI TKA ensure an increased quality of life 5‐ and 10‐years postoperatively in an aging population. From a hospital perspective, PSI TKA is less cost‐effective at 5‐ and 10‐years postoperative. Since PSI TKA is not proven to be significantly clinically superior to CI TKA, and it is more costly per patient case, its use should be considered carefully.

## AUTHOR CONTRIBUTIONS

Isobel M. Dorling was responsible for the data analysis and wrote the manuscript. Dieuwertje M.J. Theewen provided aid with the writing process and obtaining data for analysis. Ghislaine A.P.G. van Mastrigt aided the data analysis process. Tim J.M. Welting, Martijn G.M. Schotanus and Bert Boonen provided a supervisory role.

## CONFLICT OF INTEREST STATEMENT

The authors declare no conflicts of interest.

## ETHICS STATEMENT

Please include the name of the institutional review board (IRB) and the approval number. If not applicable, please state so: ABR registration number: NL31469.096.10.

## Data Availability

None declared.
